# Self-Biased Electrospun Triaxial Ferrite-PZT Nanofibers and Studies on Magneto-Electric Coupling

**DOI:** 10.3390/nano16181130

**Published:** 2026-09-10

**Authors:** Sabita Acharya, Aruna Bidthanapally, Sumayya Begum, Rao Bidthanapally, Sujoy Saha, Peng Zhou, Ovijit Das, Menka Jain, Michael R. Page, Gopalan Srinivasan

**Affiliations:** 1 Physics Department, Oakland University, Rochester, MI 48309, USA; sabitaacharya@oakland.edu (S.A.); bidthana@oakland.edu (A.B.); 94sumayya@gmail.com (S.B.); burao@oakland.edu (R.B.); 2 Department of Basic Sciences, School of Sciences and Humanities, SR University, Warangal 506371, India; sujoy.saha@sru.edu.in; 3 School of Materials Science and Engineering, Hubei University, Wuhan 430062, China; 4 Department of Physics, University of Connecticut, Storrs, CT 06269, USA; 5 Materials and Manufacturing Directorate, Air Force Research Laboratory, Wright-Patterson Air Force Base, Dayton, OH 45433, USA; michael.page.16@us.af.mil

**Keywords:** ferrite, ferroelectric, PZT, nanofibers, magnetoelectric effect

## Abstract

This work is on magneto-electric (ME) interactions in multiferroic composites of electro-spun triaxial nanofibers composed of lead zirconate titanate (PZT), strontium ferrite, SrFe_12_O_19_, (SrM), and nickel ferrite NiFe_2_O_4_ (NFO). M-type hexagonal ferrite, SrM, with a high uniaxial magneto-crystalline anisotropy field, was chosen to achieve a self-magnetic bias, and NFO with high magnetostriction and piezomagnetic coefficient was chosen to strengthen the ME coupling in the composites. By integrating these three distinct ferroic phases, the triaxial design optimizes interfacial strain transfer to enhance the ME coupling strength in the absence of an external magnetic bias. Fibers with PZT core (Sample A), SrM core (Sample B), or NFO core (Sample C) were synthesized and annealed at 750 C for crystallization of the ferroic phases. X-ray diffraction and electron and scanning probe microscopy confirmed the production of continuous, defect-free fibers with well-defined boundaries and coexisting crystalline phases free of impurities. Magnetic, ferroelectric, and magnetostrictive characterization verified ferroic ordering across all samples. Measurements of the ME voltage coefficients (MEVC) were carried out at low frequencies and at electromechanical resonance (EMR) on rectangular platelets of the fibers. A strong zero-bias ME response was measured, indicating an efficient strain-mediated coupling that bypasses the need for magnetic bias fields. Sample-C with NFO core, PZT inner shell, and SrM outer shell showed the highest low-frequency MEVC of 26.7 mV/cm Oe, and that increased to 161 mV/cm Oe at EMR, both values at zero bias. The stacking order of the core and shell phases dictated the resulting ME interactions. The results indicate the potential of these triaxial fibers as candidates for miniature magnetic sensors and arrays, multifunctional devices, and energy harvesters.

## 1. Introduction

Mechanical strain-mediated magneto-electric (ME) effects in multiferroic composites consisting of ferromagnetic and ferroelectric/piezoelectric phases have been studied extensively for the past two decades [1,2,3]. Composites with rare-earth and transition-metals and alloys, spinel and hexagonal ferrites, and manganites for the ferromagnetic phase and PZT, PMN-PT quartz, and aluminum nitride for the piezoelectric phase were studied in the past [1,2,3,4,5,6,7]. Several studies focused on a variety of applications for ME materials, including magnetic field sensors, magnetic imaging arrays, energy harvesters, and high-frequency signal processing devices [4,5,6,7]. The primary challenge, in particular, for magnetic sensors is efficient conversion of weak magnetic signals into measurable electrical outputs by enabling magnetostriction-induced deformation in the ferromagnet to translate directly into an electrical voltage through piezoelectric effects. The strength of the ME coupling relies entirely on the magnitudes of the piezomagnetic and piezoelectric coefficients and the efficient transfer of this interfacial strain. Consequently, the ME voltage coefficient (α_ME_) is highly sensitive to interfacial bonding, phase connectivity, mechanical contact, and spatial distribution. The composite structure—whether particulate, thin film, laminated, fiber-based, or core–shell—largely governs the efficiency of this strain transfer and the strengths of ME coupling [1,2,3,4,5,6,7].

Past efforts to optimize ME interactions mainly focused on various composite geometries. Particulate composites are rather straightforward to fabricate, but often suffer from randomized phase distributions, poor electrical resistivity, and, in general, a leakage current and subsequent reduction in ME coupling strengths [1,2]. Layered composites offer expansive contact areas, high resistivity, and robust ME responses, particularly near electromechanical resonance [1,8,9]. By contrast, nanocomposites such as core–shell particles and coaxial fibers provide a high surface-area-to-volume ratio, continuous morphology, and exceptionally short strain-transfer paths—making them ideal for miniaturized ME devices [2,3,10,11].

Electro-spun multiferroic nanofiber composites are the main focus of this study [3,12]. While various techniques, such as template synthesis, self-assembly, and atomic layer deposition, can generate multilayered nanostructures, electrospinning is the most versatile and scalable technique [13,14]. The technique is widely used for the fabrication of coaxial and triaxial fibers for applications in diverse fields, such as drug delivery, photocatalysis, and environmental remediation [15,16,17]. Investigations into ferrite-ferroelectric and related one-dimensional multiferroic systems demonstrate that ferrite chemistry, fiber geometry, and phase mapping heavily influence the final ME voltage coefficient [18,19,20,21,22,23]. For instance, fibers containing nickel ferrite or nickel-zinc ferrite and PZT exhibit strong ME effects due to direct core–shell contact [12,21]. Cobalt ferrite, CoFe_2_O_4_ (CFO)—PZT core–shell nanofibers were among the earliest examples of electrospun one-dimensional multiferroic fibers exhibiting ME behavior [24,25,26]. Subsequent investigations reported magnetic-field-assisted assemblies of spinel ferrite-ferroelectric fibers, coaxial hexaferrite-ferroelectric nanofibers, and studies on direct ME and magnetodielectric effects [10,11,12,21].

The vast majority of reported efforts so far on ME nanofibers, however, were restricted to two-phase core–shell geometries. Triaxial fibers were studied for applications in drug delivery and dental applications [27,28]. Starr et al. reported on the synthesis of triaxial fibers of CFO and barium titanate (BTO) [29], but ME effects in multiferroic fibers with a core–shell-shell structure are yet to be studied. Such fibers could accommodate three distinct functional phases, enhance the active interfaces, and allow for strengthening of ME effects. Here we report on the synthesis of triaxial fibers consisting of two magnetic oxides, M-type strontium hexaferrite, SrFe_12_O_19_ (SrM) and nickel ferrite, NiFe_2_O_4_ (NFO), and ferroelectric PZT. SrM, due to a high uniaxial magnetocrystalline anisotropy, can contribute to an internal magnetic bias, whereas NFO exhibits stronger magnetostrictive behavior. PZT serves as the piezoelectric phase that converts transferred strain into an electrical signal. The choice of SrM and NFO for the magnetic phases was to facilitate a built-in bias in the fibers either due to the large uniaxial crystalline anisotropy in SrM or the exchange-spring effects in SrM-NFO fibers leading to a large remnant magnetization in the fibers [30,31,32,33,34]. Thus, the novelty of this study lies in the use of phase-sequence engineering within a three-phase triaxial nanofiber. The aim is to combine the magnetic anisotropy of SrM with the magnetostrictive contribution of NFO, thereby enhancing both the low-frequency and resonance-assisted ME coupling at zero-bias.

Here we report results of our studies on the synthesis of triaxial fibers with (i) PZT-core and inner and outer shells of SrM and NFO, respectively (Sample-A), (ii) SrM-core and PZT and NFO shells (Sample-B), and (iii) NFO core and PZT and SrM shells (Sample-C) by electrospinning. Structural characterization by x-ray diffraction (XRD) and electron and scanning probe microscopies revealed the absence of impurity phases, well-formed cores and shells, and defect-free interfaces. Magnetization and ferromagnetic resonance (FMR) provided evidence for a built-in magnetic bias in the fibers. The fibers were pressed into pellets for the ME voltage coefficient measurements that indicated a significant ME response under zero-bias both at low-frequency and at electromechanical resonance.

## 2. Experiment

The procedure for the synthesis of triaxial fibers of PbZr_0.52_Ti_0.48_O_3_ (PZT), SrFe_12_O_19_ (SrM), and NiFe_2_O_4_ (NFO) involved the following steps: (i) preparation of the sol-gels, (ii) fiber synthesis by electrospinning with the use of a multi-syringe pump and a triaxial needle, and (iii) annealing of the fibers at 750 °C. The annealing temperature of 750 °C was selected based on optimization studies and previous reports to ensure complete crystallization of the constituent phases while maintaining the fibrous morphology. The first and foremost step is the preparation of individual nickel ferrite NiFe_2_O_4_ (NFO) precursor solution (sol), strontium hexaferrite SrFe_12_O_19_ (SrM) precursor sol, and lead zirconate titanate PbZr_0.52_Ti_0.48_O_3_, (PZT) precursor sol. The NFO sol was prepared by first dissolving 1.520 g of polyvinylpyrrolidone (PVP, MW = 1,300,000, Sigma-Aldrich, St. Louis, MO, USA) in a mixed solvent consisting of 13 mL ethanol (Pharmco, Dearborn, MI, USA) and 7 mL deionized water. The solution was magnetically stirred at 70 °C for 1 h until a clear and homogeneous solution was obtained. Subsequently, 1.59 g of nickel(II) acetate tetrahydrate [Ni(CH_3_COO)_2_·4H_2_O, Alfa Aesar, Ward Hill, MA, USA] and 5.24 g of iron(III) nitrate nonahydrate [Fe(NO_3_)_3_·9H_2_O, Alfa Aesar] were added simultaneously to the solution. The resulting mixture was continuously stirred at room temperature for 20 h to obtain a uniform NFO precursor sol [35]. In this formulation, PVP accounted for 6 wt.% of the total solution composition. The SrM precursor sol was prepared following a similar procedure [12]. Initially, 1.564g of PVP was dissolved in a solvent mixture of 13 mL ethanol and 7 mL deionized water, then stirred magnetically at 70 °C for 1 h until a transparent solution formed. Subsequently, 0.3006 g of strontium nitrate [Sr(NO_3_)_2_, Alfa Aesar] and 6.95 g of iron(III) nitrate nonahydrate [Fe(NO_3_)_3_·9H_2_O, Alfa Aesar] were added to the solution. The mixture was then stirred continuously at room temperature for 20 h, yielding a homogeneous SrM precursor sol. PVP constituted 6 wt.% of the total solution.

The PZT precursor sol was prepared by dissolving 3.793 g of lead acetate trihydrate [Pb(CH_3_CO_2_)_2_·3H_2_O, 99%, Alfa Aesar] in 4 mL of 2-methoxyethanol (Alfa Aesar) and refluxing at 70 °C for 1 h under continuous stirring. In a separate vessel, 1.703 g of zirconium(IV) n-propoxide (Alfa Aesar) and 1.364 g of titanium(IV) isopropoxide were dissolved in a mixture of 0.2 g of acetic acid and 4 mL of 2-methoxyethanol to form the zirconium-titanium precursor solution. This solution was added to the lead precursor solution, and the resulting mixture was refluxed at 80 °C for 9 h to obtain a homogeneous precursor. Meanwhile, 1.2 g of PVP was dissolved in a solvent mixture containing 3 mL of ethanol and 9 mL of methanol (Pharmco). The PVP solution was then incorporated into the PZT precursor solution and stirred continuously at room temperature for 20 h, yielding a homogeneous PZT sol suitable for electrospinning [35].

For triaxial fiber synthesis, we followed a similar electrospinning procedure used for fabricating nanofibers for diverse applications [36,37,38,39,40]. Our setup consisted of a multi-channel syringe pump (New Era Pump Systems), a triaxial stainless-steel needle (rame-hart), and a high-voltage power supply (Stanford Research Systems). The NFO, PZT, and SrM precursor sols were loaded into three separate 10 mL syringes and delivered to the triaxial needle at a flow rate of 0.4 mL/h. A DC voltage of 19 kV was applied between the needle and a grounded aluminum drum collector positioned 15 cm from the needle tip. Electrospinning was performed with the triaxial needle positioned horizontally and facing the grounded rotating aluminum drum collector. The process was carried out under ambient laboratory conditions at a temperature of approximately 22–25 °C and a relative humidity of 25–30%. These electrospinning parameters, including the applied voltage, flow rate, tip-to-collector distance, temperature, and humidity, were optimized to produce continuous, bead-free triaxial fibers and were maintained for the fabrication of all three samples. The sol gets electrically charged, forms a Taylor cone, and triaxial fibers are formed once the electrostatic forces overcome the sol surface tension. As the jet travels toward the collector, it undergoes continuous stretching and whipping instabilities while the solvent gradually evaporates, ultimately producing continuous core–shell-shell nanofibers. The fibers are deposited on the rotating aluminum drum. As-spun fibers were collected and dried at room temperature for 24 h and subsequently annealed in air at 750 °C for 1 h. Both the heating and cooling rates were maintained at 1 °C min^−1^.

Three different triaxial fiber architectures were fabricated to investigate the influence of phase distribution on the structural, magnetic, and magnetoelectric properties. Sample A consisted of a PZT core, SrM intermediate shell, and NFO outer shell, (PZT-SrM-NFO), whereas Sample B was composed of an SrM core, PZT intermediate shell, and NFO outer shell (SrM-PZT-NFO). In Sample C, NFO formed the core, PZT the intermediate shell, and SrM the outer shell (NFO-PZT-SrM). The crystal structure and phase composition of the fibers were characterized using X-ray diffraction (XRD, MiniFlex, Rigaku, Tokyo, Japan). Fiber morphology and microstructure were examined by scanning electron microscopy (SEM, JSM-6510GS, JEOL, Tokyo, Japan), while fiber topography and magnetic force microscopy were done using a scanning probe microscope (AFM workshop, Hilton Hill, SC, USA, Model B-3 AFM). Ferroelectric properties were measured using a Radiant ferroelectric tester. Physical Properties Measurement system (PPMS, Quantum Design Inc., San Diego, CA, USA). The saturation magnetization (M_s_) was obtained from the M-H measurements. The measured magnetization (emu/g) was converted to volume magnetization (emu/cm^3^) using the experimentally determined bulk density of the rectangular composite specimens (5.4 g/cm^3^). The resulting 4πM_s_ values were used in the Kittel fitting. FMR measurements were carried out on thin rectangular platelets of the fibers placed in an S-shaped coplanar waveguide. A 67 GHz vector network analyzer (Agilent, Santa Clara, CA, USA) was employed to record the scattering matrix S_21_ vs. frequency f profiles for a series of H applied parallel to the sample plane. Also, magnetostriction λ parallel to the in-plane static field H was measured on thin rectangular platelets using a strain gage and a strain indicator. The ferroelectric nature of the fibers was investigated by polarization P vs. E measurements. A (radiant) ferroelectric tester was used.

Magnetoelectric (ME) characterization was carried out by measuring the ME voltage coefficient (MEVC) at low frequency as a function of an applied field H and as a function of the frequency f of the AC magnetic field h. The collected nanofibers were mixed with a suitable binder and compacted into rectangular platelets and annealed for the ME measurements. For low-frequency and resonance ME measurements, rectangular samples with dimensions of approximately 9 mm × 3 mm × 0.4 mm were prepared. The samples were mounted in an aluminum sample holder that served as an effective radio frequency (RF) shield to minimize noise pickup during measurements. The measurement setup consisted of an electromagnet for applying a static magnetic field (H) and a pair of Helmholtz coils positioned inside the sample holder to generate an alternating magnetic field (h). The ac voltage induced across the sample was detected using a lock-in amplifier. The ME voltage coefficient (MEVC) was measured as a function of H and f.

## 3. Results

### 3.1. Structural Characterization

**Figure 1:**
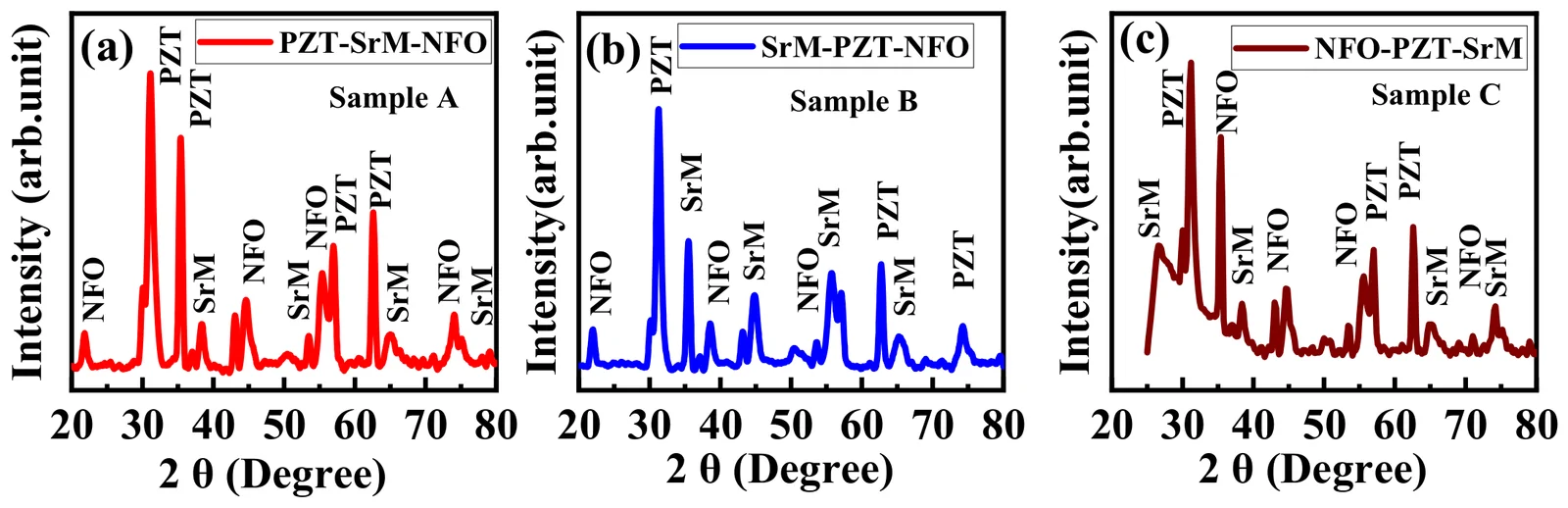
X-ray diffraction (XRD) data for annealed triaxial fibers of (**a**) Sample A, (**b**) Sample B, and (**c**) Sample C (NFO-PZT-SrM).

Results of the X-ray diffraction (XRD) measurements for the annealed core–shell–shell fibers are presented in Figure 1. The diffraction patterns for Samples A, B, and C exhibit characteristic reflections corresponding to NFO, PZT, and hexagonal SrM phases. The indexed diffraction peaks, including PZT (110), (200), (211), and (220), NFO (220), (311), (422), (422), (533), and (440), and SrM (201), (116), (2010), (2011), (224), (406), (217), (220) and, (008) confirm the formation of all three constituent phases after annealing without detectable secondary or impurity phases. The absence of impurity peaks indicates that the electrospinning and subsequent heat-treatment process preserves the phase purity of each constituent despite the complex triaxial architecture.

Although the fibers have identical phases, differences in the relative diffraction peak intensities are observed because the core–shell-shell configuration changes the volume fraction and spatial distribution of each phase within the fibers. Sample A exhibits relatively stronger SrM reflections, whereas Sample B shows more pronounced PZT peaks. In contrast, Sample C displays comparatively stronger NFO reflections, particularly the (311) peak. For all three samples, the PZT diffraction peaks are sharper than those of NFO and SrM, indicating higher crystallinity and larger crystallite size of the PZT phase. This observation is consistent with previous reports on ferrite-PZT composites, where the PZT phase exhibits well-defined diffraction peaks due to its superior crystal ordering and grain growth during annealing [41,42].

**Figure 2:**
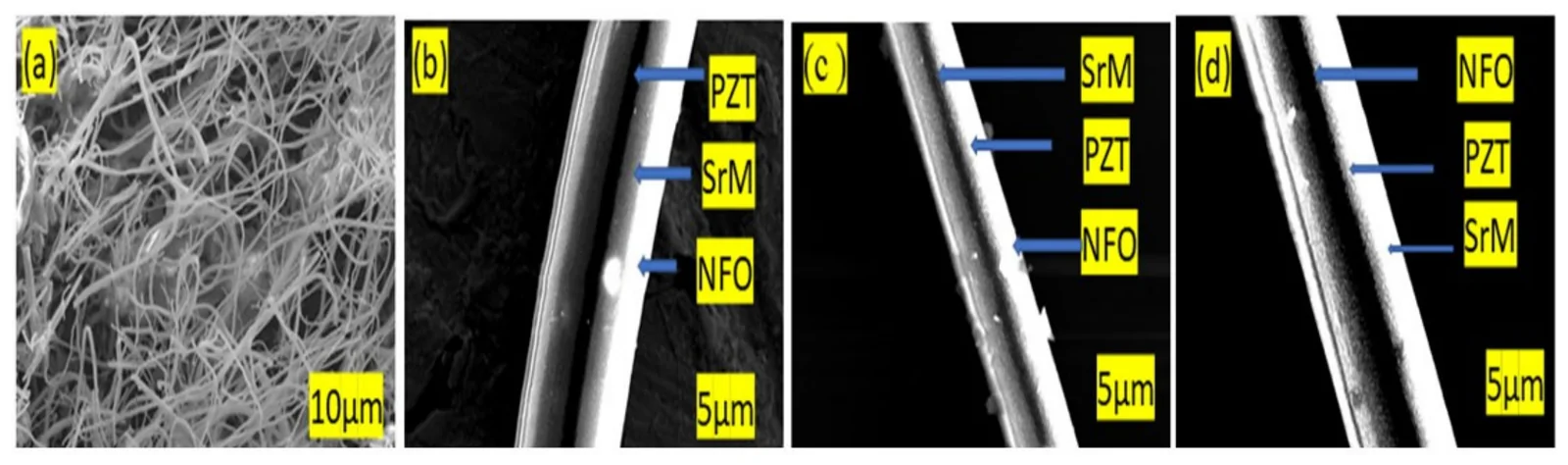
Scanning electron microscopy (SEM) images of (**a**) a collection of fibers of Sample A, (**b**) a fiber of Sample A, (**c**) a fiber of Sample B, and (**d**) a fiber of Sample C.

The annealed fibers at high temperature were imaged by scanning electron microscopy (SEM) and are shown in Figure 2. The low-magnification SEM image in Figure 2a shows a continuous, randomly entangled network of electrospun fibers with relatively uniform diameters. We have quantified the fiber morphology by measuring the diameters using ImageJ 1.54r software for Windows. A significant fraction of the fibers were found to have diameters in the range 6–9 μm. The higher-magnification images in Figure 2b–d display only short sections of individual fibers to reveal the triaxial core–shell-shell architecture. The apparent discontinuity of some fibers arises from the limited SEM field of view and occasional fracture during post-annealing handling and sample preparation, rather than from discontinuous fiber formation during electrospinning. The fibers have lengths ranging from approximately 10 to 30 μm. Based on the triaxial electrospinning configuration, Sample A was designed to contain a PZT core surrounded by SrM and NFO intermediate and outer shells, respectively; Sample B was designed with an SrM core and PZT and NFO intermediate and outer shells; and Sample C was designed with an NFO core and PZT and SrM intermediate and outer shells. SEM-EDS analysis confirmed the presence of the constituent elements associated with the three phases, namely Pb, Zr, and Ti for PZT, Sr for SrM, and Ni for NFO, together with Fe and O. However, because the overall fiber diameter is only a few thousand nanometers while the interaction volume of SEM-EDS at 15–20 kV is typically ~0.5–2 μm, the EDS signal contained contributions from all three layers. Therefore, SEM-EDS confirmed the coexistence of the three constituent phases within the fibers but did not provide their chemical composition. Verification of the radial chemical distribution may require cross-sectional TEM characterization. The diameter of the PZT core was estimated to be approximately 200 nm. In sample B, the core is composed of the ferromagnetic SrM phase, while the intermediate and outer shells consist of PZT and NFO, respectively. The diameter of the SrM core was found to be approximately 100 nm. In sample C, the triaxial structure consists of an NFO core, a PZT intermediate shell, and an outer SrM shell. The diameter of the NFO core is found to be 200 nm. The distinct core–shell-shell morphologies observed in all three samples confirm the successful fabrication of triaxial nanofibers with different phase arrangements. Our previous studies on coaxial ferrite-ferroelectric fibers indicated the presence of fibers with diameters ranging from 100 nm to 500 nm and 60% of the fibers with diameters in a narrow range [3,12]. The fiber dimensions depend on the viscosity of the sols, the magnitude of the electric field applied, and the humidity in the electrospinning chamber [12].

**Figure 3:**
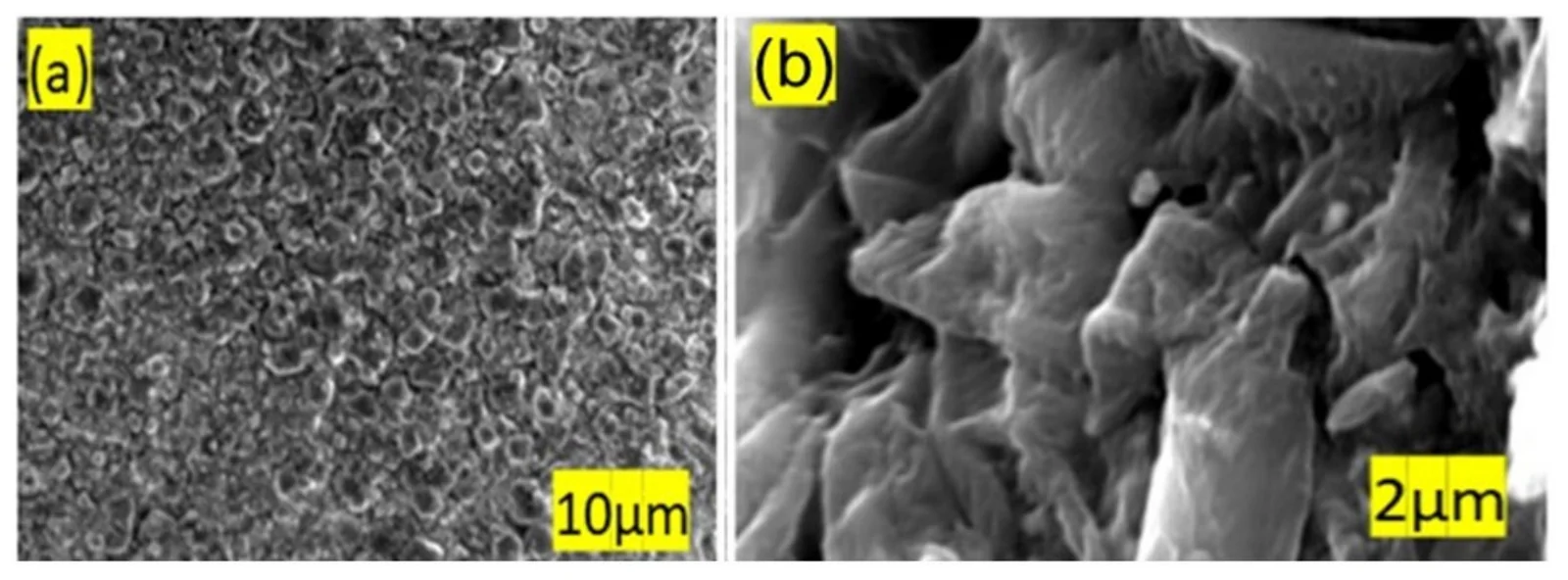
SEM micrographs of a pressed and annealed platelet of fiber A at different magnifications.

We also imaged the surface profile of rectangular platelets of the fibers prepared for magnetic and MEVC measurements. SEM images at different magnifications in Figure 3 show interconnected fibrous morphology for the samples. The microstructure remains distinctly fibrous rather than transforming into a randomly dispersed particulate morphology. These observations indicate that the electrospun fiber network is largely preserved after compaction at high pressure and annealing at high temperature.

**Figure 4:**
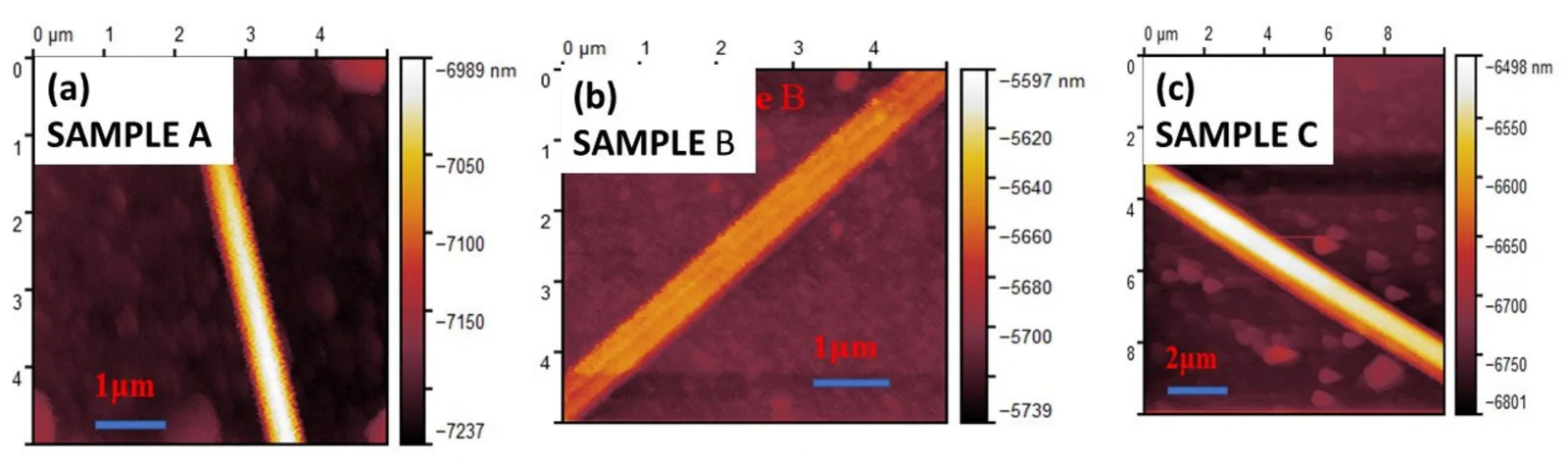
Atomic force microscopy (AFM) topography of (**a**) Sample A (PZT-SrM-NFO), (**b**) Sample B (SrM-PZT-NFO), and (**c**) Sample C (NFO-PZT-SrM).

**Figure 5:**
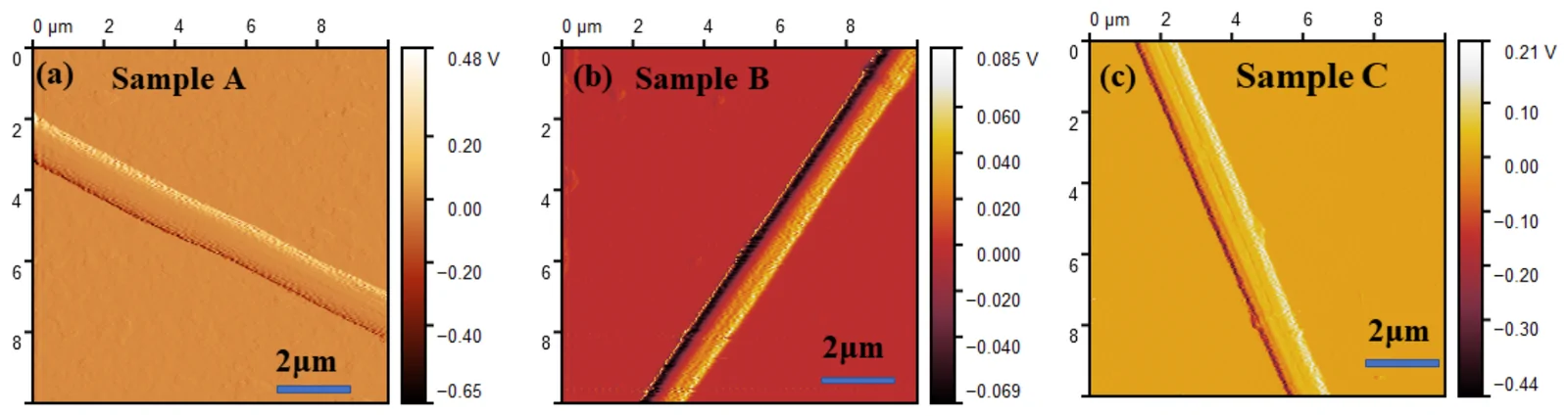
Magnetic force microscopy (MFM) phase image for (**a**) sample A (PZT-SrM-NFO), (**b**) Sample B (SrM-PZT-NFO), and (**c**) Sample C (NFO-PZT-SrM).

The fibers were characterized using atomic force microscopy (AFM) and magnetic force microscopy (MFM). The AFM topography images of Samples A, B, and C are shown in Figure 4a–c, respectively, while the corresponding MFM phase images are presented in Figure 5a–c. AFM measurements were performed to examine the topography. Sample A exhibits a continuous fiber-like feature with a clear height contrast relative to the surrounding surface. Sample B shows a continuous fiber extending diagonally across the scanned region, with comparatively lower height contrast and several localized surface features. Sample C exhibits a well-defined continuous fiber with a pronounced height contrast and localized raised features on the surrounding surface. These results demonstrate the continuous fiber-like surface morphology of all three triaxial configurations, with variations in the surface features and height distribution depending on the layer sequence. The corresponding MFM phase images exhibit distinct magnetic contrast along the fibers, indicating magnetic response associated with the fiber structures.

### 3.2. Magnetic Characterization

**Figure 6:**
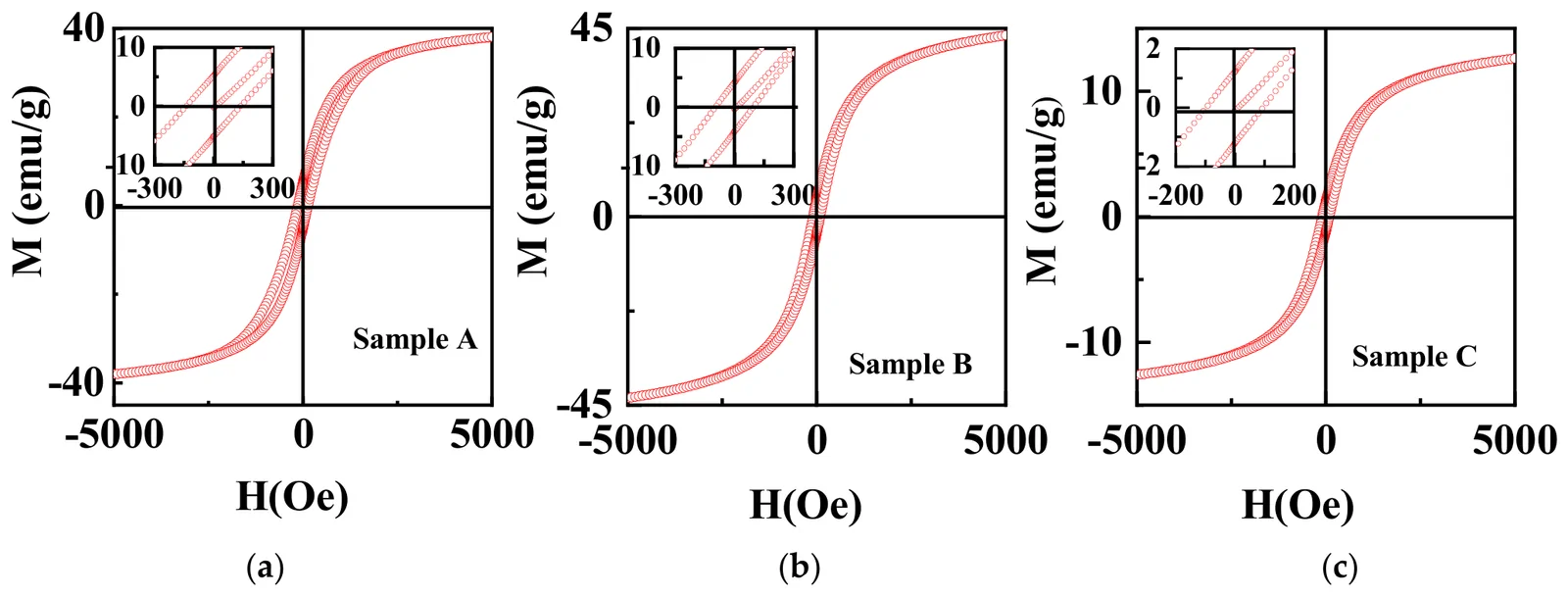
Room-temperature magnetic hysteresis (M–H) loops of the triaxial nanofibers: (**a**) PZT–SrM–NFO (Sample A), (**b**) SrM–PZT–NFO (Sample B), and (**c**) NFO–PZT–SrM (Sample C). The insets show enlarged views of the low-field region used to determine the remanent magnetization (M_r_) and coercive field (H_c_).

For magnetic characterization, including magnetization, FMR, and magnetostriction measurements, the fibers were compacted into thin rectangular specimens and subsequently annealed at 750 °C. The magnetization M as a function of applied static magnetic field H was measured using a Physical Property Measurement System (PPMS, Quantum Design Inc., San Diego, CA, USA) and is shown in Figure 6. It shows the room-temperature magnetic hysteresis (M-H) loops of the triaxial nanofibers with different layer sequences: PZT-SrM-NFO (Sample A), SrM-PZT-NFO (Sample B), and NFO-PZT-SrM (Sample C). All samples exhibit well-defined ferromagnetic hysteresis loops, with magnetization increasing rapidly at low fields, confirming ferromagnetic ordering in the triaxial structures. Expanded views near the origin (insets) reveal narrow hysteresis loops characteristic of low-coercivity magnetic behavior. Sample A exhibits the highest remanent magnetization (M_r_ = 5 emu/g and coercive field H_c_ = 137 Oe), followed by Sample B (M_r_ = 4.2 emu/g, H_c_ = 92 Oe), while Sample C shows the lowest remanent magnetization (M_r_ = 1.2 emu/g) with a comparable coercivity (H_c_ = 91 Oe). Although all three triaxial nanofibers contain identical magnetic constituents (SrM and NFO), the measured magnetic parameters vary with the layer sequence. Sample A exhibits the highest remanent magnetization and coercive field, whereas Sample C shows the lowest remanent magnetization. The observed variation in M_r_ and H_c_ indicates that the radial architecture influences the magnetic behavior of the triaxial nanofibers, despite their identical phase composition.

**Figure 7:**
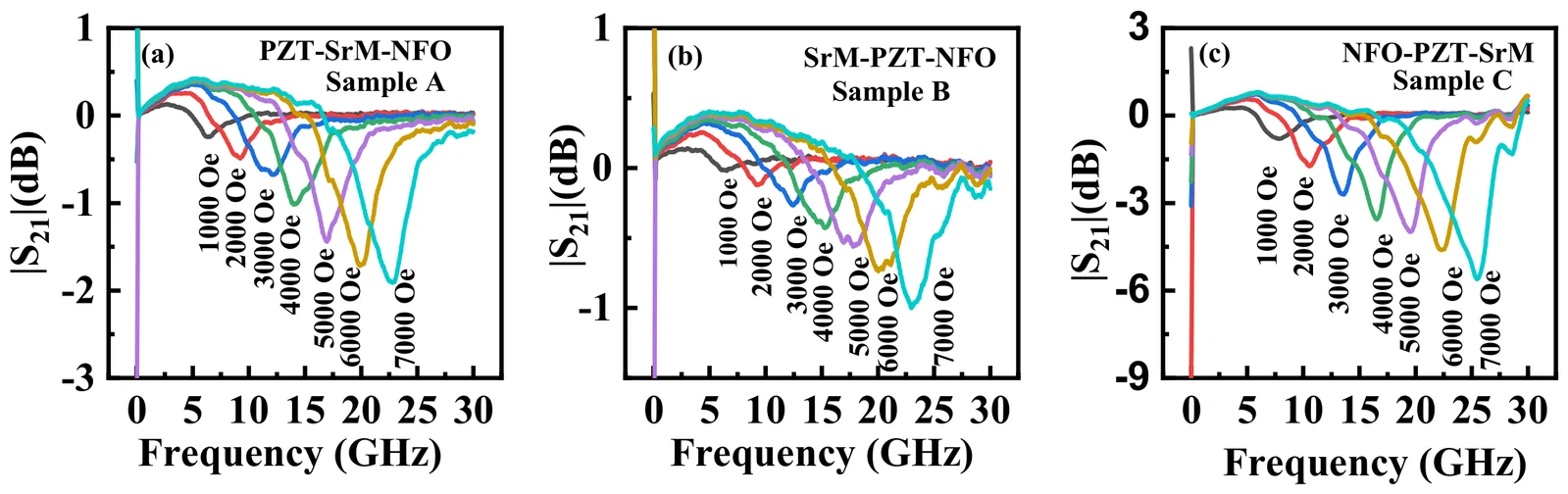
Scattering matrix parameter S_21_ vs. frequency f for ferromagnetic resonance (FMR) in Sample A (**a**), Sample B (**b**), and Sample C (**c**).

**Figure 8:**
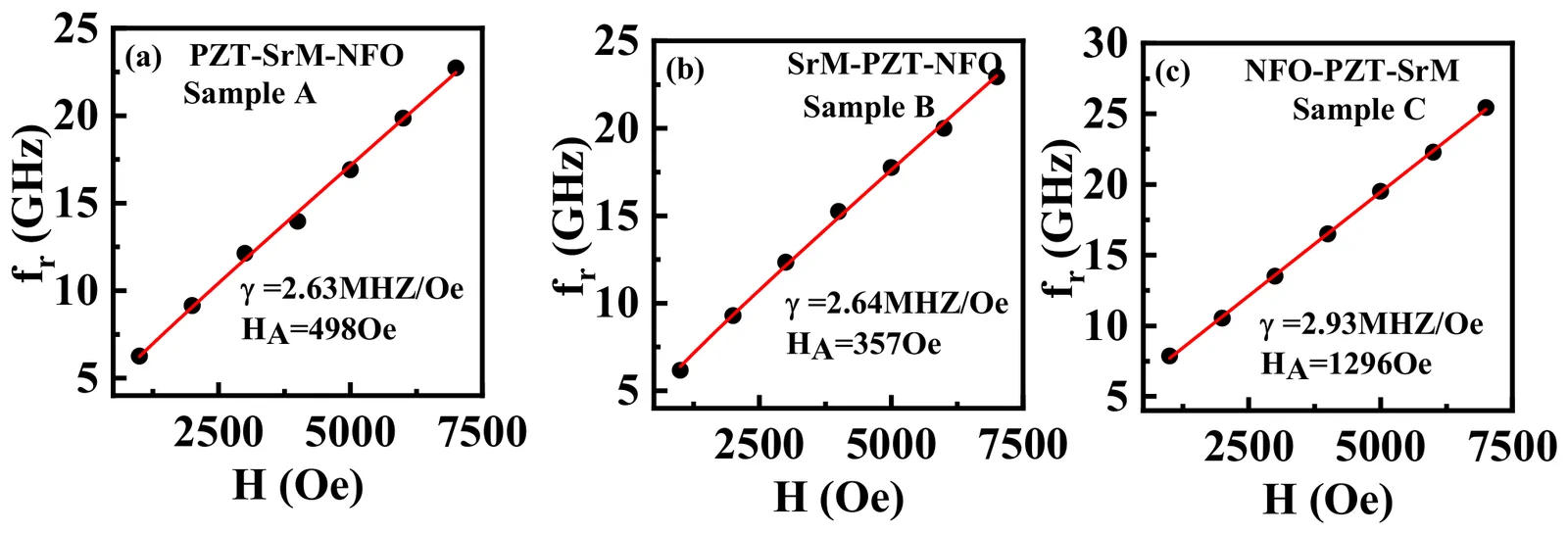
The fitting of the resonance frequency f_r_ vs. H to Kittel’s equation to determine the gyromagnetic ratio γ and the anisotropic field H_A_ for Sample A (**a**), Sample B (**b**), and Sample C (**c**).

FMR measurements on thin rectangular platelets of the fibers were done with the sample in a coplanar waveguide and for the static field H parallel to the sample plane. Profiles of the scattering matrix parameter S_21_ vs. f obtained with a VNA for the samples are shown in Figure 7 and Figure 8. The FMR manifests as a sharp dip in the profiles and it shifts to higher frequencies with increasing H.

In addition, the intensity of microwave absorption increases with increasing H. At the same time, the resonance peaks become progressively narrower, indicating reduced magnetic damping and improved coherence of spin precession at higher fields. These observations confirm the presence of well-defined FMR modes in the triaxial nanofiber composites and demonstrate the strong influence of the applied magnetic field on their dynamic magnetic response.

Data on the resonance frequency f_r_ as a function of H were obtained from the FMR profiles in Figure 6 and are shown in Figure 7. The gyromagnetic ratio γ and the magnetocrystalline anisotropy field H_A_ could then be determined by fitting the f_r_ vs. H data to the Kittel equation for FMR for in-plane H given by:

f_r_ = γ [(H + H_A_) (H + H_A_ + 4πM_s_)]^1/2^ (1)

Values of 4πM_s_ were determined from M-H measurements in Figure 5 after converting the magnetization from emu/g to g/cm^3^ using the experimentally measured bulk density of 5.4 g/cm^3^ for all three samples A, B, and C, The calculated γ values for the triaxial fibers were found to range from 2.63 to 2.93 MHz/Oe, which are slightly lower than 3 MHz/Oe reported for nickel ferrite and hexaferrites such as BaM [43,44]. The estimated magneto-crystalline anisotropy H_A_ ranged from 357 Oe for Sample-B with SrM core to 1296 Oe for Sample-C with SrM outer shell.

**Figure 9:**
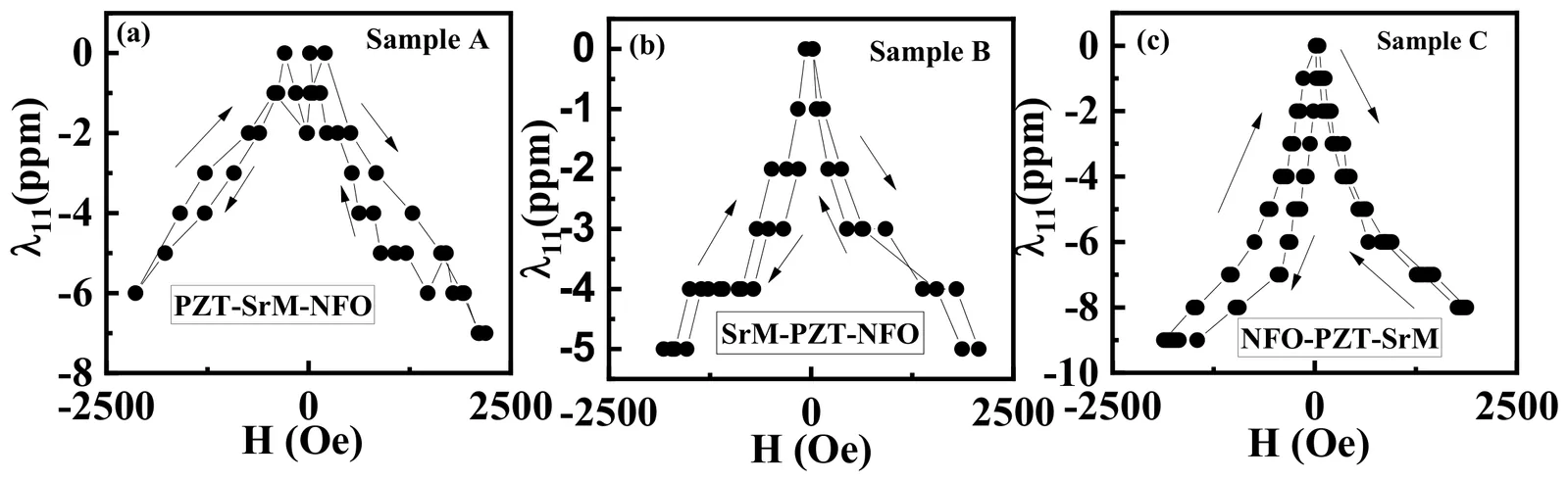
Magnetostriction λ_11_ measured parallel to the applied in-plane magnetic field H for (**a**) samples A, (**b**) Sample B, and (**c**) Sample C. The arrows indicate increasing or decreasing H direction.

Magnetostriction is a key parameter that determines the strength of ME coupling in the composites. The magnetostriction measurements were carried out on platelets prepared from the fiber samples. A strain gage was bonded to the sample surface with a thin layer of a two-part epoxy adhesive (M-Bond 600, Micro-Measurements, Wendell, NC, USA). The magnetostriction λ_11_ parallel to in-plane H was measured as a function of H. Figure 9 shows λ_11_ vs. H data for all nanofiber composites. As expected, the magnetostriction is negative and its magnitude increases with increasing H. It is clear from the data that there is significant variation in the magnitude of λ_11_ with the nature of core–shell-shell phase sequence in the fibers. Sample C (NFO-PZT-SrM) shows the highest λ_11_ value of −9 ppm, followed by Sample A (PZT-SrM-NFO), while Sample B (SrM-PZT-NFO) shows the lowest value of −5 ppm.

### 3.3. Ferroelectric and Magneto-Electric Characterization

**Figure 10:**
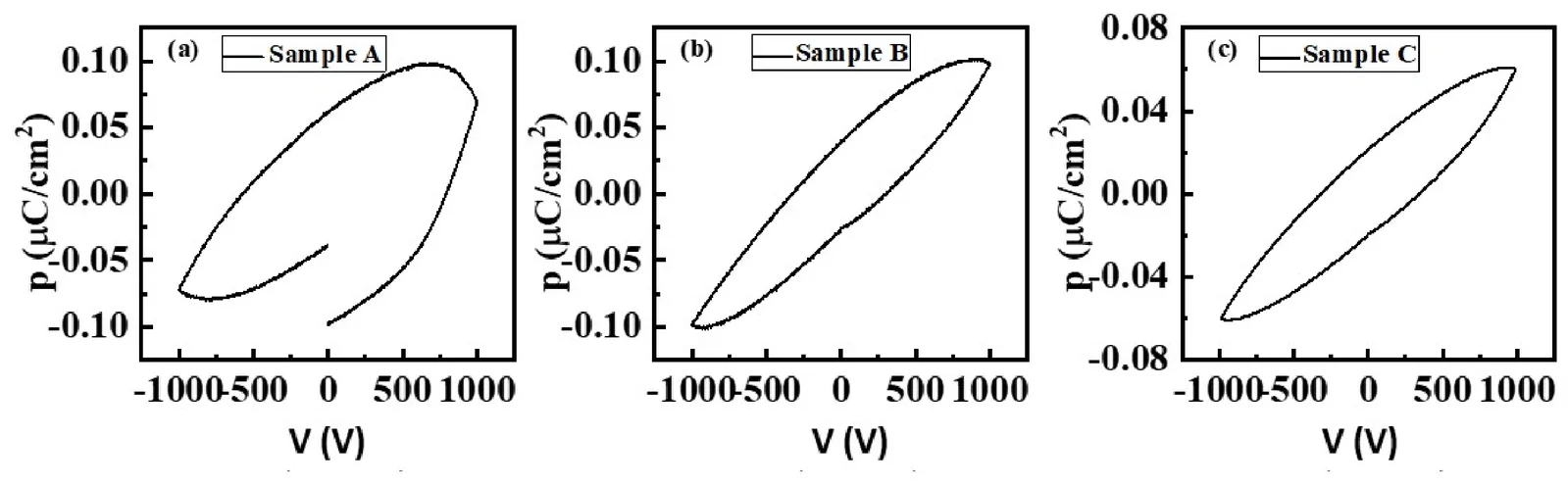
Ferroelectric Polarization P as a function of voltage V for samples A (**a**), B (**b**), and C (**c**).

Figure 10 shows the P-V hysteresis loops of the triaxial nanofiber composites measured with a ferroelectric tester on platelets of the fibers. All samples exhibit ferroelectric behavior, confirming the contribution of the PZT phase. The loops are not fully saturated, which is commonly observed in nanostructured ferroelectric composites due to leakage currents, porosity, grain boundaries, and incomplete domain alignment. Sample A shows the broadest hysteresis loop and the highest polarization values, suggesting stronger ferroelectric activity and a larger switchable polarization. Sample B exhibits a narrower and slimmer hysteresis loop than Sample A, indicating a comparatively weaker ferroelectric response and lower hysteretic energy loss. Sample C shows the smallest polarization values and the slimmest loop, although ferroelectric switching is still evident. These results confirm that the core–shell-shell phase sequence affects the electrical behavior of the fibers.

**Figure 11:**
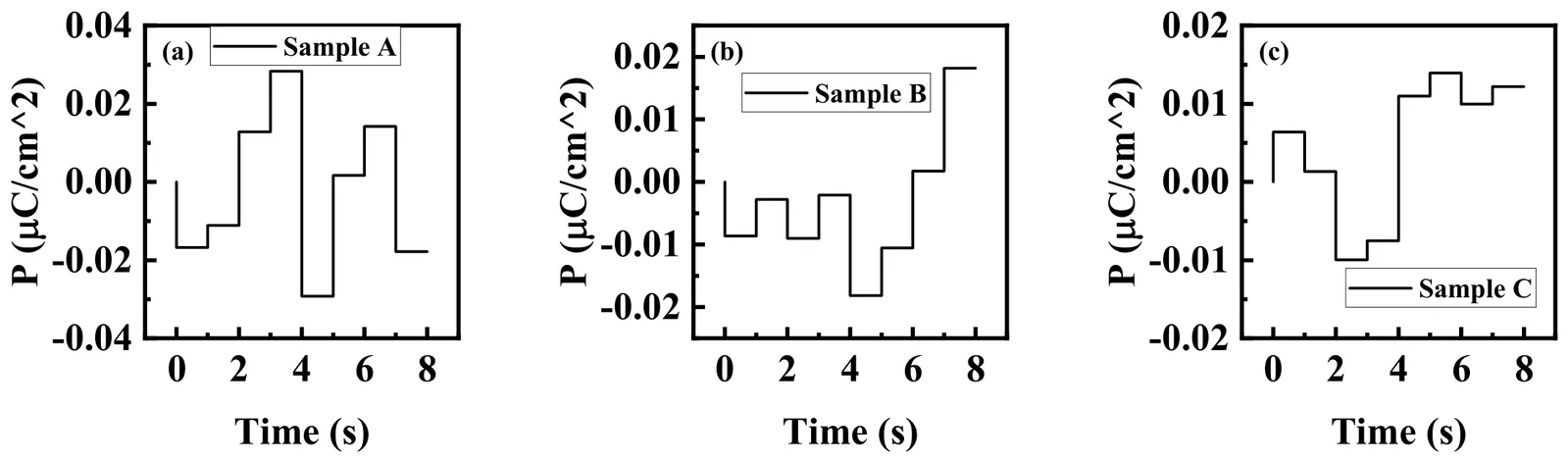
PUND (positive up negative down) polarization versus time for a series of 500 V pulses of duration 1 s for Sample A (**a**), Sample B (**b**), and Sample C (**c**).

To further distinguish the switchable ferroelectric polarization from non-switching contributions such as leakage current and dielectric charging, positive up negative down (PUND) measurements were performed on all triaxial nanofiber composites. Distinct polarization changes are observed in Figure 11 during the PUND pulse sequence for all samples, confirming the presence of switchable ferroelectric polarization. Compared with the P-V hysteresis loops, the PUND measurements help distinguish the switchable polarization from non-switching contributions, such as leakage current and dielectric charging. However, the PUND responses are not perfectly symmetric between the positive and negative pulses, indicating that polarization reversal is incomplete. This asymmetric behavior is likely caused by internal bias fields at the ferrite/PZT interfaces, residual polarization from the pre-poled state of the samples, and limited domain switching under the applied electric field. Therefore, the observed asymmetry does not indicate the absence of ferroelectricity; rather, it suggests that the polarization switching is influenced by interfacial effects and domain pinning in the triaxial nanofiber composites.

The magnitude of the switchable polarization follows the order Sample A (PZT–SrM–NFO) > Sample C (NFO–PZT–SrM) > Sample B (SrM–PZT–NFO), which is generally consistent with the overall trend observed in the P vs V hysteresis measurements. Sample A exhibits the largest polarization response, suggesting more effective participation of the PZT phase in the switching process, whereas Samples B and C show reduced polarization because the different triaxial phase arrangements modify the electric-field distribution, ferroelectric phase connectivity, and interfacial mechanical constraints.

Even though all samples demonstrate ferroelectric switching behavior, their measured polarization values are lower than those of dense bulk PZT ceramics. This reduction makes sense for electrospun nanocomposites because of their nanoscale structure, the small volume fraction of the PZT phase, and the internal interfaces between the PZT and ferrite phases. The alignment between the switchable polarization from PUND and the P–V hysteresis behavior shows that the observed polarization mainly comes from reversible ferroelectric domain switching, not just from leakage or interfacial polarization. The varying polarization levels among the samples result from their different core–shell-shell configurations, which affect the electric-field distribution, PZT phase connectivity, and the mechanical limits set by the surrounding magnetic layers. Thus, the PUND findings add weight to the idea that the arrangement of phases significantly affects the ferroelectric response of triaxial nanofiber composites.

For the magnetoelectric (ME) measurements, the electrospun triaxial nanofibers were first annealed at 750 °C, compacted into rectangular-shaped specimens using a small amount of binder, and subsequently re-annealed at 750 °C. As discussed in the SEM analysis (Figure 3), the binder-pressed specimen retains an interconnected fibrous morphology. Consequently, the measured ME response is attributed to interactions within the retained fibrous network, where efficient strain transfer between the piezoelectric PZT phase and the magnetic ferrite phases is facilitated through the interconnected fiber architecture. Both the static magnetic field H and the AC field h were applied along the length of the sample (direction-1), and the strain-induced AC voltage V was measured across its thickness (direction-3). The H-dependence of the ME voltage coefficient α_31_= V/(h t), where t is the sample thickness, is shown in Figure 10, and the results are for a frequency of 100 Hz for h and for both increasing and decreasing values of H. One may infer the following from the data in Figure 10. (i) The most significant observation is the large zero-bias MEVC for all three samples that arises due to a self-bias in the fibers. (ii) The strength of ME coupling shows a pronounced dependence on the contents of the fibers, with the highest MEVC for the fibers in which the PZT shell is sandwiched between the NFO core and SrM shell. The lowest MEVC is measured in fibers with SrM core and PZT and NFO inner and outer shells, respectively. (iii) A general decrease in the magnitude of α_31_ with H occurs in all the samples. (iv) A hysteresis is seen in the MEVC vs. H data for all the fibers.

**Figure 12:**
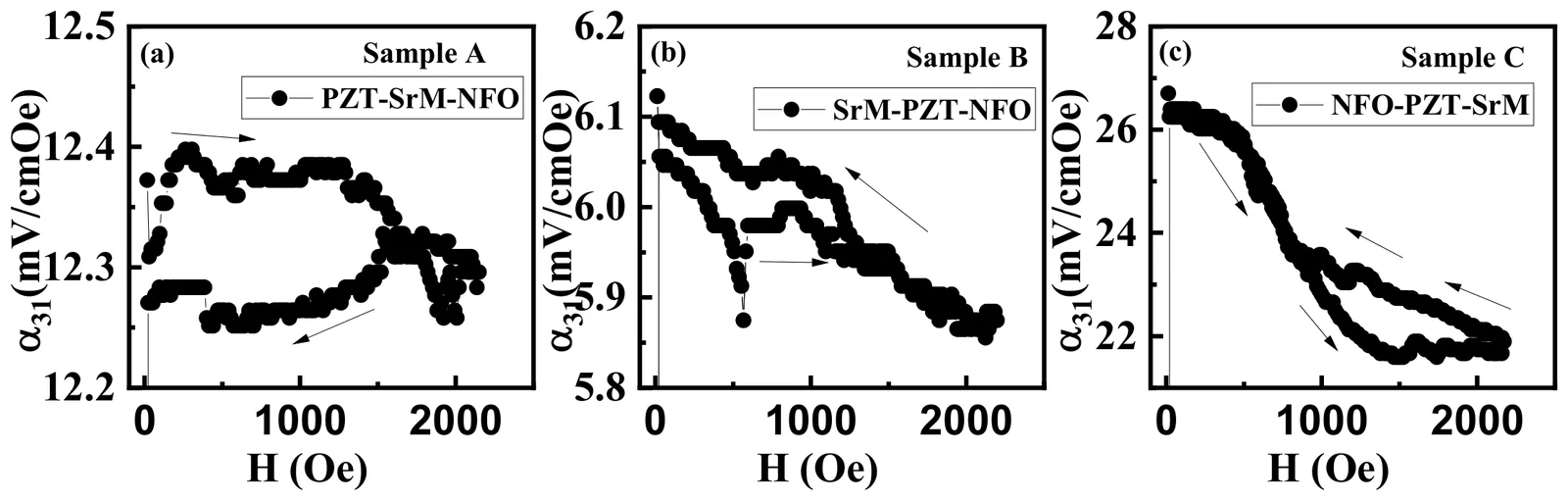
ME voltage coefficient α_31_ measured at 100 Hz as a function of the bias field H for Samples A (**a**), B (**b**), and C (**c**). The arrows indicate increasing or decreasing H direction.

**Figure 13:**
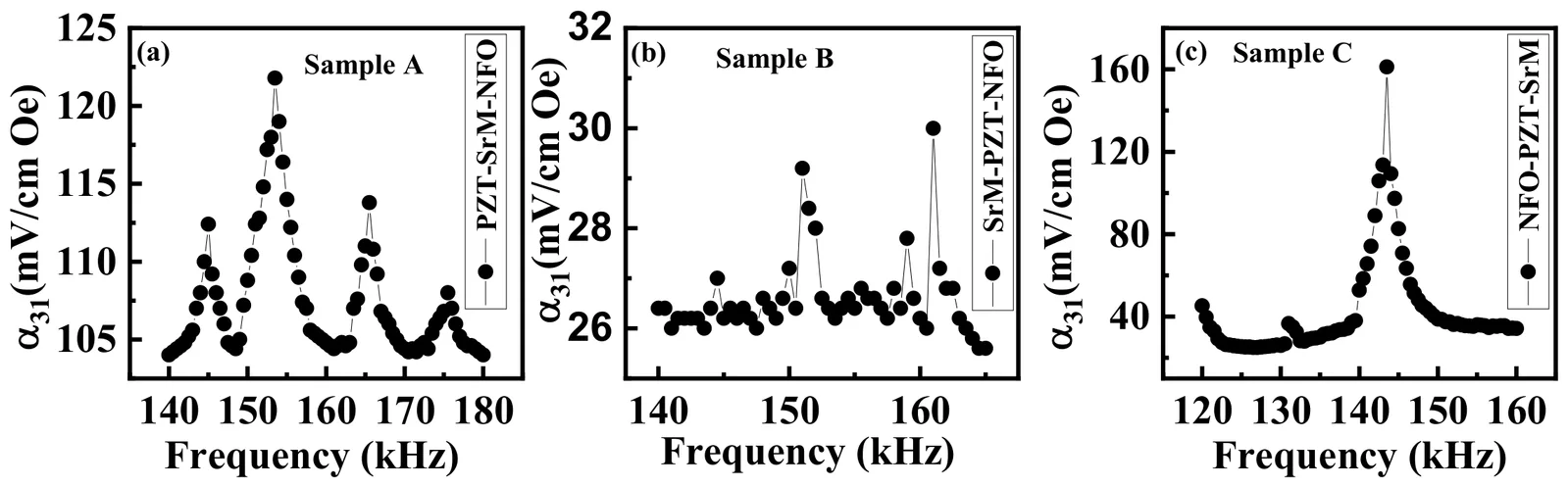
Frequency dependence of ME coefficient α_31_ for Samples A (**a**), B (**b**), and C (**c**).

Figure 12 shows the dependence of MEVC on the frequency f of the AC magnetic field for samples A, B, and C. Since the MEVC in Figure 12 is maximum under zero-bias, the measurements were made under zero external bias. All the samples show resonance enhancement of MEVC at overall frequencies f_r_ = 120–180 kHz, corresponding to longitudinal electro-mechanical resonance mode (EMR) in the samples that depends on the sample dimensions. A maximum MEVC occurs at f_r_, and the secondary peaks in Figure 13a,b are most likely due to a minor nonuniformity in the sample dimensions. Sample A shows a resonance MEVC of 121 mV/cm Oe at 153 kHz, whereas Sample B shows a much lower value, ~30 mV/cm Oe at 161kHz. The strongest resonance enhancement is observed for Sample C with a value of 161 mV/cm Oe at 143 kHz. Overall, both field-dependent and frequency-dependent measurements confirm that the NFO-PZT-SrM architecture provides the most effective core–shell-shell arrangement for near-zero-bias and resonance-enhanced magnetoelectric coupling.

## 4. Discussion

The triaxial core–shell-shell fibers composed of nickel ferrite (NFO), strontium ferrite (SrM), and PZT were synthesized by electrospinning and then annealed at high temperatures, which was essential for crystallizing the ferroic oxides. X-ray diffraction confirmed the formation of the desired crystalline phases, with no detectable impurity phases. Scanning electron microscopy and scanning probe microscopy revealed fibers with uniform diameter and well-defined core, middle, and outer shells.

The assignments of the core, intermediate, and outer layers are based on the predetermined triaxial electrospinning configuration, in which the corresponding precursor sols were introduced through the inner, intermediate, and outer channels of the triaxial needle. Cross-sectional TEM combined with STEM-EDS mapping of course could provide the most direct verification of the radial chemical distribution in the triaxial fibers. Since TEM characterization was not available for the present study, SEM-EDS analysis was performed to confirm the presence of the constituent elements associated with the three phases, namely Pb, Zr, and Ti (PZT), Sr (SrM), and Ni (NFO), along with Fe and O. We further note that the overall fiber diameter is only a few thousand nanometers, whereas the SEM-EDS interaction volume at typical accelerating voltages of 15–20 kV is approximately 0.5–2 μm, which exceeds the fiber diameter. Consequently, the EDS signal contains contributions from all three layers, making it difficult to resolve the individual shells or unambiguously determine the radial sequence by SEM-EDS alone. Although the exact chemical compositions could not be determined, the XRD data did not reveal any impurity phases in the annealed fibers.

The ferromagnetic nature of the fibers was confirmed through magnetization and FMR measurements. Data on M vs. H revealed typical ferromagnetic behavior and saturation of M for H > 2 kOe. Although the fibers had both NFO and SrM, the very low remnant magnetization M_r_ and coercive field indicate an exchange-coupled ferromagnetic phase with characteristics similar to that of NFO. One may infer from the low M_r_ values that there is a lack of evidence for the exchange-spring effects reported in nanocomposites of hexagonal ferrite-spinel ferrite [31,32,33]. The gyromagnetic ratio γ for the fibers was found to be in the range of 2.63–2.93 MHz/Oe, which is lower than the reported values of 3.0–3.3 MHz/Oe for bulk nickel ferrite and SrM. Among the investigated samples, the SrM-PZT-NFO triaxial nanofibers exhibited the highest saturation magnetization of 43.33 emu/g and the largest magnetic anisotropy field obtained for NFO-PZT-SrM 1296 Oe. The magnetostriction measurements showed that the maximum value obtained in the present work was approximately −9 ppm for the NFO-PZT-SrM fibers. This value is lower than the previously reported magnetostriction of −12 ppm for coaxial fibers consisting of a PZT core and a nickel-zinc ferrite shell [21]. Both values are considerably smaller than those reported for polycrystalline pure nickel ferrite and nickel-zinc ferrite [34,45], which can be due to the composite architecture and the presence of the non-magnetostrictive piezoelectric phase.

The ferroelectric polarization-voltage (P-V) measurements confirmed the ferroelectric behavior of the fibers. However, the measured polarization values were lower than the 2–3 μC/cm^2^ reported for NFO-BTO fibers [12]. The reduced polarization is likely due to increased leakage current and associated dielectric losses in the composite fibers. The most significant aspect in the present study is the strength of ME coupling. The present study demonstrates a maximum zero-bias magnetoelectric (ME) voltage coefficient of 26.7 mV/cm Oe for the fibers with NFO core, PZT inner shell, and SrM outer shell, which compares favorably with many previously reported ferrite-piezoelectric fiber systems. Randomly distributed NZFO-PZT composite fibers exhibited ME coefficients of only 3–4 mV/cm Oe [21] while NFO-BTO fibers showed values of approximately 0.5 mV/cm Oe [12]. Similarly, NFO-hexagonal ferrite fibers have been reported to exhibit ME coefficients in the range of 12–24 mV/cm Oe [3]. Thus, the triaxial NFO-PZT-SrM fibers developed in this work provide a substantially enhanced zero-bias ME response compared with these previously reported systems.

The present work demonstrates success with our approach of combining a hexagonal ferrite and spinel ferrite in a nanocomposite to achieve significant zero-bias coupling. Unlike conventional coaxial fibers that contain a single magnetic component, the triaxial configuration creates multiple magnetostrictive–piezoelectric interfaces, enabling more efficient strain transfer and stronger interfacial coupling. Furthermore, the coexistence of soft magnetic NFO and hard magnetic SrM, each possessing different magnetic anisotropy and magnetostriction characteristics, can generate complementary magnetic interactions that contribute to an enhanced self-biased magnetoelectric response.

The strong ME coupling strength in Sample C with NFO core and PZT and SrM shells is consistent with high magnetostriction and confirms that the radial arrangement of SrM, PZT, and NFO strongly affects the near-zero-bias ME behavior of the triaxial nanofiber composites. An additional and significant advantage of the triaxial architecture is revealed by the frequency-dependent ME measurements. Sample C exhibits the highest zero-bias ME voltage coefficient of 161 mV/cm Oe under EMR, which is a factor of six higher than the low-frequency zero-bias value of 26.7 mV/cm Oe. This substantial enhancement indicates highly efficient ME coupling at EMR.

Thus, the novelty of the present work is the successful realization of a triaxial core–shell–shell nanofiber platform that integrates multiple functional interfaces and dual magnetic phases to achieve a large zero-bias ME response. These results suggest that triaxial nanofibers represent a promising strategy for the development of self-biased ME sensors, energy harvesters, and low-power multifunctional devices, where eliminating the need for an external DC bias field is highly desirable.

## 5. Conclusions

Multiferroic triaxial nanofibers consisting of a core, an inner shell, and an outer shell were fabricated by electrospinning. Two ferrimagnetic oxides, NFO with high magnetostriction and SrM with very high magneto-crystalline anisotropy, were chosen to obtain a self-biased ferromagnetic phase with a large magnetostriction, and ferroelectric PZT. Fibers with cores of PZT, NFO, or SrM were made and annealed at high temperatures. Structural characterization revealed the absence of impurity phases and defect-free fibers of uniform radial dimensions and well-defined interfaces. Saturation magnetization for the fibers ranged from 10 to 43 emu/g. FMR measurements provide evidence for a large magnetocrystalline anisotropy H_A_ in the fibers that could only be attributed to the strontium hexagonal ferrite. H_A_ is lowest for Sample B with SrM as the core and a slight increase in H_A_ is measured for Sample A with SrM in the inner shell. Sample C with SrM as the outermost shell shows the highest H_A_. One anticipates H_A_ to depend critically on the volume fraction of SrM, which depends on several factors including (i) the dimension of the core and shell in the triaxial needle, (ii) the sol viscosity, and (iii) the dimension of the core and shells in the fibers. Studies indicate a wide distribution in the dimensions of the core and shells in the fibers. It is somewhat difficult to attribute H_A_ to the volume fractions of SrM since its volume fraction in the fibers is unknown. However, the present study does indicate that a large H_A_ for the fibers with SrM outer shells. Magneto-electric voltage coefficients were measured at low frequencies and at EMR on rectangular platelets of the fibers. Strong ME coupling under zero bias was evident for all fibers. Samples with fibers of NFO core, PZT inner shell, and SrM outer shell showed the highest zero-bias MEVC of 26.7 mV/cm Oe at 100 Hz and a resonance-enhanced value of 161 mV/cm Oe at 143 kHz. These results highlight the effectiveness of using a ferromagnetic composite for self-biased multiferroics. The phase-sequence engineering approach in this study is found to enhance strain-mediated coupling and demonstrate the potential of triaxial nanofibers for magnetic sensing, energy harvesting, spintronic devices, and other multifunctional magnetoelectric technologies.

## Data Availability

The data generated and/or analyzed during the current study are available from the corresponding author upon reasonable request.
